# Arenaviruses and Lethal Mutagenesis. Prospects for New Ribavirin-based Interventions

**DOI:** 10.3390/v4112786

**Published:** 2012-11-06

**Authors:** Héctor Moreno, Ana Grande-Pérez, Esteban Domingo, Verónica Martín

**Affiliations:** 1 Centro de Biología Molecular “Severo Ochoa” (CSIC-UAM), Campus de Cantoblanco 28049, Madrid, Spain; Email: hmoreno@cbm.uam.es (H.M.); edomingo@cbm.uam.es (E.D.); 2 Área de Genética, Facultad de Ciencias, Campus de Teatinos, Universidad de Málaga, 29071, Málaga, Spain; Email: agrande@uma.es; 3 Centro de Investigación Biomédica en Red de Enfermedades Hepáticas y Digestivas (CIBERehd), 08036 Barcelona, Spain; 4 Centro de Investigación en Sanidad Animal (CISA), Carretera de Algete a El Casar s/n, 28130 Valdeolmos, Madrid, Spain; Email: veronica.martin@inia.es

**Keywords:** viral quasispecies, lymphocytic choriomeningitis virus, 5-fluorouracil, antiviral treatment

## Abstract

Lymphocytic choriomeningitis virus (LCMV) has contributed to unveil some of the molecular mechanisms of lethal mutagenesis, or loss of virus infectivity due to increased mutation rates. Here we review these developments, and provide additional evidence that ribavirin displays a dual mutagenic and inhibitory activity on LCMV that can be relevant to treatment designs. Using 5-fluorouracil as mutagenic agent and ribavirin either as inhibitor or mutagen, we document an advantage of a sequential inhibitor-mutagen administration over the corresponding combination treatment to achieve a low LCMV load in cell culture. This advantage is accentuated in the concentration range in which ribavirin acts mainly as an inhibitor, rather than as mutagen. This observation reinforces previous theoretical and experimental studies in supporting a sequential inhibitor-mutagen administration as a possible antiviral design. Given recent progress in the development of new inhibitors of arenavirus replication, our results suggest new options of ribavirin-based anti-arenavirus treatments.

## 1. Introduction: complexity of LCMV populations

Michael B.A. Oldstone wrote that “LCMV has proven to be a Rosetta stone for uncovering numerous phenomena in virology and immunology”. The several crucial achievements were listed by Oldstone in an introduction to an outstanding compilation of review articles on arenavirus biology [1]. Yet, arenaviruses pose important challenges regarding the mechanism of replication and regulation of gene expression of a segmented genome, as well as disease mechanisms, prevention and treatment. Persistent arenavirus infections are highly prevalent among rodents, and when some of the viruses infect humans they may cause severe disease, including hemorrhagic fevers [2,3,4]. The prototypic arenavirus LCMV is increasingly regarded as a neglected human pathogen [5,6,7,8]. Unfortunately, the number of preventive or therapeutic possibilities for arenavirus-associated disease is very restricted. Despite promising developments [9,10,11,12], no licensed vaccines are generally available, and current therapy is essentially limited to an off-label use of the antiviral agent ribavirin (1-*β*-D-ribofuranosyl-1-*H*-1,2,4-triazole-3-carboxamide) (Rib) [13,14]. This is why arenavirus-related diseases are an interesting target to explore new antiviral designs.

Lack of effective vaccines and antiviral treatments is a general difficulty for diseases associated with highly variable RNA viruses, due to quasispecies dynamics [15]. The root of the problem is that mutations occur at such a high rate during RNA genome replication that even what is generally termed the “wild type” virus is, at any time, an average of a multitude of sequences. Although our attention is often fixed on a consensus sequence or on specific mutants identified in mutant spectra, in reality we deal with mutant clouds of rather indeterminate composition despite transient dominance of some specific mutant residues. Analyses of mutant spectra by ultra-deep sequencing have confirmed the extreme complexity of viral populations in nature, in support of previous evidence obtained by molecular cloning and sequencing ([16,17], among other examples [15]). LCMV participates of high mutation rates and quasispecies dynamics, with a direct implication in viral persistence and disease ([18,19,20,21,22,23,24,25,26,27,28,29,30,31,32]; reviews in [33,34]). The range of mutation frequencies calculated with genomes sampled from mutant spectra of LCMV populations passaged in cell culture in absence of mutagenic agents is in the range of 1.0 × 10^-4^ to 7.5 × 10^-4^ substitutions per nucleotide (s × nt^-1^), and mutation frequencies reached 3.6 × 10^-3^ and 1.1 × 10^-3^ s × nt^-1^ when the virus was passaged in the presence of 5-fluorouracil (FU) or Rib, respectively [35,36,37,38,39,40]. Mutation frequencies are in agreement with values of Shannon entropy, a measure of the proportion of different sequences in a genome distribution. Both parameters are used to quantify the complexity of mutant spectra, which is influenced by the number of passages from a clonal origin (i.e. starting with a plaque-purified virus), the multiplicity of infection (MOI), and the viral gene analyzed. Several studies indicate that the complexities of mutant spectra of LCMV are comparable to those determined for other RNA viruses [15].

## 2. Contributions of LCMV to lethal mutagenesis

Mutations often entail a fitness cost, and this applies also to drug-escape mutants. The almost systematic selection of drug-resistant viral mutants can be explained by the existence of multiple pathways to resistance, some with limited fitness cost (under a genomic sequence context or a given environment), and to the selection of compensatory (fitness-enhancing) mutations in viral genomes that continue expressing the resistance phenotype [15]. Under this scenario, it has been amply recognized that new antiviral strategies must be investigated to avoid or minimize the selection of drug or multi-drug-escape mutants, and the consequent treatment failure. Strategies already implemented or under development are combination therapies, splitting of treatment between an induction and a maintenance regimen, use of drugs that target cellular functions, and combination of immunotherapy and chemotherapy. As reviewed in [15], none of these strategies is free of the problem of selection of escape mutants. 

An additional strategy now under investigation is lethal mutagenesis, also termed virus entry into error catastrophe, in recognition of its conceptual origins. It consists in achieving large reductions of viral load and ideally virus extinction by increasing the mutation rate of the virus above a critical error rate that sets a limit for virus viability. Current research involves the use of mutagenic base or nucleoside analogues which are converted intracellularly in the nucleoside-triphosphate derivatives which are incorporated into viral RNA during elongation. They do not act as chain terminators, a mechanism that results mainly in inhibition, but they allow continued chain elongation, a mechanism that results in mutagenesis. The latter is a result of the ambiguous pairing with the corresponding complementary purine or pyrimidine nucleotides, when the analogue is either a substrate or a template residue [41]. Lethal mutagenesis was inspired in the concept of crossing an error threshold, or transition into error catastrophe, established as an important corollary of quasispecies theory [42,43]. Crossing the error threshold implies loss of genetic information. It was reasoned that drugs that increase the mutation rate of a virus above a viability threshold may highly diminish the chances of virus escape. Mutagenesis can provide an advantage over conventional non-mutagenic inhibitors because mutagenized genomes may suppress the most infective subpopulations of genomes coexisting in the same quasispecies, as discussed in the next section. A number of base and nucleoside analogues are currently under development as potential new antiviral mutagenic agents [44,45,46,47,48,49,50].

At present it is not known whether in the context of a clinical application of lethal mutagenesis [51], mutagen-resistant mutants will be as readily selected as inhibitor-resistant mutants. Rib-resistant mutants have been isolated in cell culture [52,53,54,55,56,57,58,59,60,61,62,63,64,65], and in patients subjected to Rib monotherapy [66]. Rib resistance can have multiple mechanisms, as evidenced by the fact that resistance mutations have been mapped in several viral genes. When resistance is associated with amino acid substitutions in the polymerase, it can be due to restoration of polymerase activity or to alterations of nucleotide recognition. The latter can occur through at least three mechanisms: average increase of template-copying fidelity, specific decrease of Rib-triphosphate incorporation (without a significant effect on general fidelity), or modulation of the transition types produced in the presence of Rib (without alteration of the average mutation frequency among the components of the mutant spectrum) [55,57,58,59,61]. 

LCMV has had an important contribution regarding the feasibility of a lethal mutagenesis-based approach *in vivo* [37], and in the proposal of the lethal defection model of virus extinction (participation of defective viruses in decrease of replicative competence of a viral quasispecies [67]), two of the highlights of lethal mutagenesis research (Table 1). In addition, early experiments evidenced that LCMV could be efficiently extinguished by enhanced mutagenesis [35] (comment by Eigen [68]). The transition towards extinction did not involve any modification of the consensus genomic sequence, and entailed a 10^2^- to 10^3^-fold decrease in specific infectivity of LCMV [36]. These were revealing observations to interpret the molecular events associated with virus extinction. 

**Table 1:** Some highlights in letahal mutagenesis research

Observation and implications	References
• J.J. Holland and colleagues explore for the first time quasispecies error catastrophe with real viruses and show that poliovirus and vesicular stomatitis virus have very limited tolerance to increased mutagenesis.	[69]
*This study was the birth of experimental studies on the application of the concept of error catastrophe developed by M. Eigen, P. Schuster and colleagues.*	
• L.A. Loeb, J. Mullins and colleagues show that a mutagenic pyrimidine analogue impairs HIV-1 replication in cell culture. They coin the term “lethal mutagenesis”.	[70]
*This study suggested the use of mutagenic agents as antiretroviral drugs.*	
• E. Domingo, P. Lowenstein and colleagues show that lymphocytic choriomeningitis virus and foot-and-mouth disease virus can be extinguished by mutagenic agents, and that low viral load and low viral fitness favor extinction.	[35,71]
*These experiments suggested that modification of virus population parameters, specifically a decrease in fitness and low viral load, may help in producing virus extinction.*	
• S. Crotty, R. Andino, C.E. Cameron and colleagues demonstrate that the antiviral ribonucleoside analogue ribavirin is mutagenic for poliovirus.	[72]
*This important discovery implied that ribavirin might be exerting some of its antiviral clinical activity as a mutagen. This is still a debated issue, but there is evidence that ribavirin is mutagenic for a number of RNA viruses including LCMV.*	
• 5-Fluorouracil impeded the establishment of a persistent LCMV infection in mice.	[37]
*This experiment constitutes a proof of principle of the feasibility of a lethal mutagenesis-based antiviral approach in vivo.*	
• Experimental and theoretical evidence for the lethal defection model of virus extinction.	[67]
*These results introduced the concept that a mutagenic agent may not only “kill” virus but that, more subtly, the agent may be generating interfering genomes that participate in the impairment of viral replication and eventual extinction. The results suggested also the possibility of guiding internal interactions within mutant spectra to achieve extinction through modest mutagenic intensities.*	
• When a mutagen participates in therapy, a sequential inhibitor-mutagen administration might have an advantage over the corresponding combination treatment.	[73,74,75]
*These studies illustrate that the interactions among drugs must be considered with regard to efficacy, in particular in the case of antiviral inhibitors used with virus-specific mutagenic agents. The general advantage of a combination therapy need not apply when a mutagen is involved in therapy.*	
• 5-Azacytidine can induce lethal mutagenesis of HIV-1	[76]
*This result suggests that some nucleotide analogues can be incorporated both into RNA and DNA during the retroviral life cycle. It shows also that there is room for classic antiviral and anti-cancer agents to find an application in lethal mutagenesis.*	
• First clinical trial involving administration of a pyrimidine analogue to AIDS patients. The resident HIV-1 was mutagenized although no virus extinction was achieved.	[51]
*In addition to representing the first clinical trial based on lethal mutagenesis, this result opens the possibility of improved efficacy in vivo using either combination or sequential administration of inhibitors and mutagens, in conjunction with provirus mobilization from carrier cells, a point under active investigation.*	

## 3. Lethal defection and the anti-arenavirus activity of ribavirin

Lethal defection introduced a role of internal interactions among components of a mutant spectrum in the process of virus extinction. The key observation was that when a persistent LCMV infection of BHK-21 cells was perturbed by addition of the mutagen FU, viral infectivity decreased at a faster rate than the level of viral RNA [67]. The unexpectedly faster kinetics of loss of infectivity than viral RNA was interpreted as due to the presence of a class of defective genomes, termed “defectors”, that were generated as a consequence of mutagenesis. Defectors could replicate their RNA but jeopardize formation of infectious particles, contributing to a decline of infectivity that was more substantial than the decline of viral RNA. The experimental findings were supported by *in silico* simulations of the outcome of LCMV replication in the absence or presence of defectors under different mutagenic intensities [67,77]. The combination of theory and experiment led to the proposal of the lethal defection model, according to which defectors play an important role in virus extinction. The term defector had been previously used to refer to other types of non-functional genomes in models of RNA virus evolution [78,79]. In lethal mutagenesis, a defector is a genome that manifests some defect during its replication cycle, that may or may not complete production of infectious particles, but that is competent in RNA replication. The latter feature is essential to express an interfering activity against fully infectious viral genomes, as documented with specific foot-and-mouth disease (FMDV) capsid and polymerase mutants displaying high or low levels or RNA replication [80]. Furthermore, an interfering, replication-competent virus with two polymerase substitutions lost its interfering activity when a third polymerase mutation that abolished RNA replication was introduced in the genome [80]. Current models suggest that interference can come about through expression of non-functional or suboptimal viral proteins by the defectors, since a majority of viral proteins are multi-functional and active through formation of homomeric or heteromeric complexes with viral or host proteins (for example, proteins that must interact to form the viral capsid or RNA replication complexes). A protein that includes an amino acid substitution at critical contact site, and that is encoded by a subset of genomes from a mutant spectrum, may contribute to formation of either non-functional or suboptimal complexes, thereby decreasing the replicative efficiency of the viral genomes that exploit that particular protein complex. In a mutagenized mutant spectrum, this mechanism may have a multiplicative effect as a result of the action of many low frequency variants harboring “defector-prone” mutations that may adversely affect one or more viral functions. This view is an extension of interference as defined in classic genetics [81], applied to a context of multitudes of micro-interference events in a mutant cloud [15].

A direct biochemical evidence of formation of suboptimal protein complexes as a result of mutagenesis is still lacking. However, interference by mutant spectra has been reported for a variety of virus-host systems in cell culture and *in vivo*, and suppression can limit replication of high fitness, virulent or drug-resistant mutants [32,67,82,83,84,85]. Internal interactions within mutant spectra confer viral quasispecies an identity as units of selection, a hallmark of the quasispecies concept [42]. In the original quasispecies theory the unit of selection was founded on the cross-talk through mutation among components of the same mutant spectrum. In viruses the cross-talk occurs through interactions among expression products. Such interactions represent an important conceptual departure over classic models that viewed quasispecies (or “intra-host variations”, as sometimes inaccurately named) as mere aggregates of mutants resulting from a mutation-selection equilibrium [15,86]. Both, in theoretical quasispecies and in viral quasispecies an interacting mutant ensemble is the unit of selection.

## 4. Sequential *versus* combination antiviral treatments

### 4.1. The advantage of combination treatments to control viral quasispecies may not apply universally

There is little question that administration of combinations of two or more drugs diminish the probability of selecting drug-escape mutants for rather obvious statistical reasons, a concept that has been amply supported by theoretical studies and clinical practice (reviewed in [15]). A similar argument applies to the control of variable infectious cellular agents such as bacteria (accentuated in those displaying a mutator phenotype), parasites or cancer cells [87,88,89].

Initial experiments on mutagenesis-based extinction of FMDV and HIV-1 showed that the addition of an antiviral inhibitor to a mutagenic agent facilitated extinction, in particular when the target viruses displayed high replicative fitness [90,91]. Since low viral load favored extinction by mutagenesis [71], the advantage of a combination of a mutagen and an inhibitor was interpreted as being a consequence of the reduction of viral load caused by the inhibitor. However, upon further examination of FMDV extinction mediated by Rib mutagenesis and inhibition by guanidine hydrochloride (an inhibitor of picornavirus RNA replication), alternative protocols were compared. They involved serial virus passages in the presence of the following drugs: (i) guanidine alone; (ii) Rib alone; (iii) a combination of guanidine and Rib; and (iv) first guanidine followed by Rib. Unexpectedly, protocol iv rendered the lowest viral loads and earliest extinction [73]. The results were supported by a theoretical model on the predicted consequences of a mutagenic agent and an antiviral inhibitor acting simultaneously or sequentially on a viral quasispecies [73]. A mutagen can display potentially conflicting activities: it may increase the frequency of defectors to favor extinction or the frequency of inhibitor-resistant mutants to hinder extinction. In addition, it was shown experimentally that the presence of guanidine, but not of Rib, prevented the interference exerted by specific FMDV mutants [73], in agreement with the fact that interfering mutants must replicate their RNA to exert interference [80]. Thus, at least on two grounds (the effects of a mutagen and an inhibitor acting on a replicating mutant spectrum and a diminished interference due to the presence of an inhibitor that prevents replication of defectors), a combined administration (simultaneous presence) of an inhibitor and a mutagen might limit the efficacy of lethal defection-mediated extinction.

Further elaboration of the theoretical model, together with additional experiments with FMDV, delimited a range of parameters (in particular the intensities of mutagenesis and inhibition) under which a sequential inhibitor-mutagen administration could have an advantage over the corresponding combination treatment [74,75]. These studies indicated that the efficacy of a sequential treatment was restricted to an inhibitor-mutagen administration (not the converse) and was more prominent when high initial inhibitory concentrations were used. In contrast, the model predicts that a combined administration is always preferred over a sequential administration when either two inhibitors or two mutagens are involved in therapy [73,74,75]. The case of two inhibitors has been amply demonstrated, and a preference of combination over sequential treatments is currently reflected in standard clinical practice. In the light of the experimental results with FMDV and the predictions of the theoretical model of viral dynamics [73,74,75], we have now explored alternative lethal mutagenesis protocols with LCMV.

### 4.2. Ribavirin in sequential *versus* combination administration for lethal mutagenesis of LCMV

**Figure 1:**
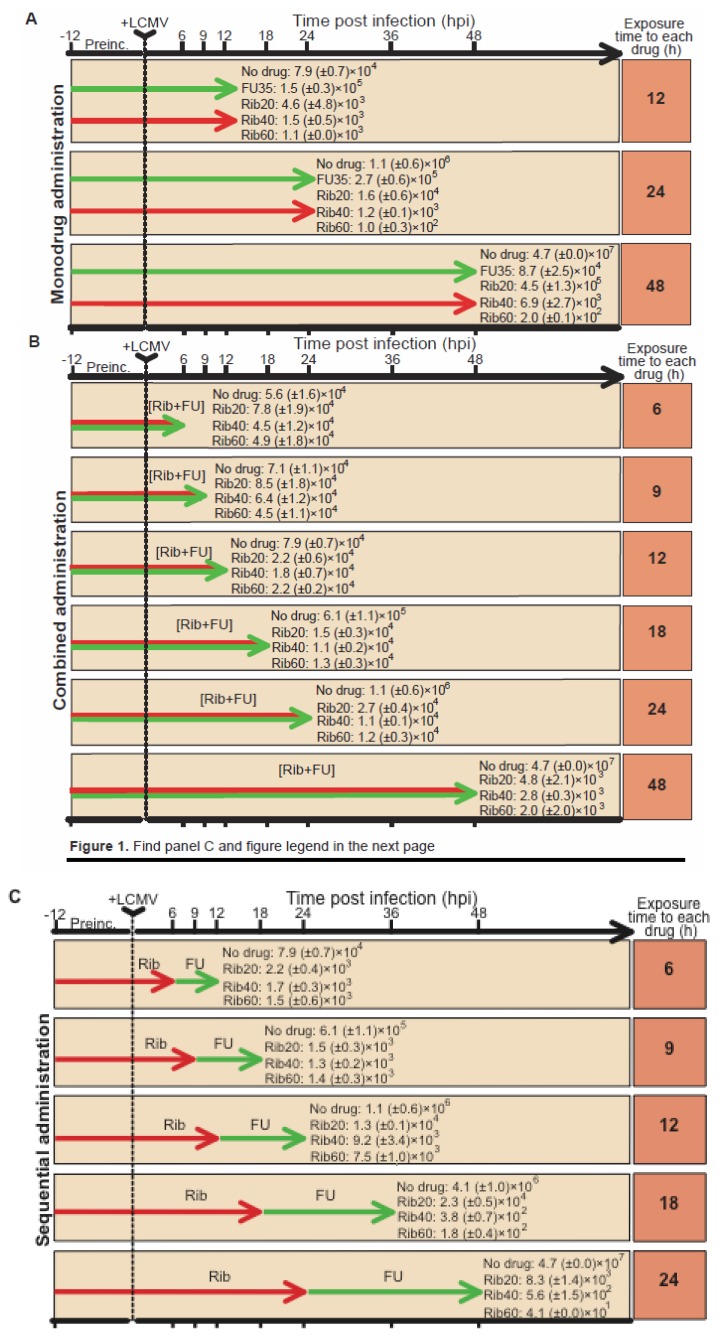
LCMV production following different drug treatments. The time of preincubation (Preinc.) with the indicated drugs, and the time of measurement (expressed in hours) of the virus titer in the cell culture supematants is indicated with the arrow at the top of each panel. +LCMV indicates infection of BHK-21 cells with LCMV Arm 53 at a multiplicity of infection of 10 PFU/cell. Titiers, obtained by plaque assay on Vero cell monolayers, are expressed in PFU/ml and are the average (+ SD) of three determinations, FU was used at 35 µg/ml, and Rib at the indicated µM concentrations, as justified in the text. “No drug” means the same infection protocol in the absence of any drug at any time. The top panel (A) corresponds to LCMV infectious progeny production following treatment with either FU or Rib alone (monotherapy). The middle panel (B) corresponds to LCMV production following combination [Rib+FU] treatments. The bottom panel (C) corresponds to LCMV production following Rib, FU sequential administrations. To change the drug in the culture medium, the medium was removed, the monolayer washed three times with medium (DMEM with 5% foetal calf serum), and the new medium with the desired drugs added to the monolayer. The middle panel gives the production following the combined [Rib + FU] administrations. The statistical significances of the differences between infectious LCMV production under different drug concentrations and administration protocols are given in the text. Toxicity of drugs for BHK-21 cells, procedures for drug treatments, LCMV infections and plaque assay have been previously described [35,36,38,39,40,56].

LCMV infection offered a unique opportunity to further test a possible advantage of a sequential inhibitor-mutagen *versus* a combination protocol since Rib displays a dual mutagenic (at low concentrations) and inhibitory (at high concentration) activity on LCMV in cell culture [39]. The efficiency of a sequential Rib → FU *versus* a combination [Rib + FU] treatment, was investigated using three Rib concentrations (20, 40 and 60 μM), and a fixed FU concentration (35 μg/ml). The range of Rib concentrations was such that its activity went from being mainly mutagenic (20 μM) to mainly inhibitory (60 μM) [39]. FU was used at a concentration which is mutagenic but permissive for continued LCMV replication [35]. Treatment with a single drug (either FU or Rib), as well as sequential and combination anti-LCMV protocols with the two drugs were compared in the course of a single infection of BHK-21 cells (without virus passage). The experimental procedures were those used in our previous studies [35,36,37,38,39]. Viral infectivity in the cell culture supernatants was determined at different hours post infection (hpi) (Figure 1). At early times (12 and 18 hpi), the sequential treatment yielded lower levels of infectious virus under all conditions tested, with a ratio of titers of sequential *versus* combined administration (measured at the same time p.i.) in the range of 0.07 to 0.12 (calculated from the data shown in Figure 1). The same ratio calculated at 24 hpi yielded a range of 0.48 to 0.62. Interestingly, an advantage (lower yield of infectious virus) of the sequential Rib (60 μM) → FU over the corresponding combination was noted at 48 hpi, with a titer ratio of 0.02 and 1.73 with 60 μM Rib and 20 μM Rib, respectively. The higher effectiveness of the Rib → FU sequential treatments occurred despite exposure to each drug for 48h during the combination treatment, and 24h during the sequential administrations (compare arrows in Figure 1B, C). The decrease in infectious virus yield at 48 hours post infection correlated with the concentration of Rib, either in monotherapy or in the combination and sequential protocols involving Rib and FU (p<0.05, Spearman correlation test). This is expected from previous studies that showed an inhibitory activity of Rib on LCMV production, that increases with Rib concentration [39]. At 48 hours post infection, a lower LCMV yield in the sequential relative to the combined administration of Rib and FU was observed only with Rib concentration of 40 μM and 60 μM, and in both cases the differences were statistically significant (p=0.0015 and p=0.0011, respectively; Mann-Whitney test). In contrast, no such advantage of the sequential treatment was observed when the Rib concentration was 20 μM (p=0.0557; Mann-Whitney test), concentration at which Rib acts as a mutagen [39]. In fact, the tendency was the contrary: with 20 μM Rib, the sequential administration produced a 1.7-fold higher LCMV yield than the corresponding combined administration (Figure 1). This is in agreement with theoretical predictions based on experimental parameters obtained with FMDV that suggest that when either two inhibitors or two mutagens (rather an inhibitor and a mutagen) are involved in therapy, a combination treatment will generally be more effective that the corresponding sequential treatment [76]. The differences in virus yield between sequential and combined administrations, measured at earlier times post infection, are also statistically significant (p<0.05; Mann-Whitney test). The decreases in infectivity were paralleled by decreases in viral RNA production (data not shown). 

The results indicate that the previously documented benefits of a sequential inhibitor-mutagen administration described with FMDV and supported by a model of virus dynamics [73,74,75], applies also to LCMV, and the advantage was more pronounced when the virus titer was measured at late time (48 hpi). It must be noted that the design of the experiment with LCMV (Figure 1) differs from the previous one with FMDV [74,75,76] in that rather than completing several passages (each of them until overt cytopathology was observed) in the presence of either an inhibitor, a mutagen or both, here we have sampled the supernatant of the same cell culture at different times post infection, as in previous protocols of lethal mutagenesis of LCMV [38,67]. The different design with FMDV and LCMV is partly justified by the cytolytic nature of FMDV infections *versus* persistence of LCMV (with no detectable cytopathology) in BHK-21 cells (compare Figure 1 with the experimental schemes reported in [73,75]).

To investigate whether Rib and FU exerted a mutagenic activity in the course of the different treatments (Figure 1), we determined the complexity of the mutant spectra of LCMV populations at 48 hpi produced under either sequential of combined drug treatments. To this aim, RNA extracted from the cell culture supernatant was subjected to RT-PCR amplification and molecular cloning under conditions of excess viral RNA template to ensure that the resulting molecular clones were a non-redundant sample of the viral genomes in the population. This procedure and its controls were carried out as detailed previously [39]. The results (Table 2) show that for each Rib concentration tested, the complexity of the mutant spectrum was significantly higher in samples subjected to sequential than to combined administration (p<0.05; χ^2^ test). The types of mutations indicate a dominance of A → G transitions during the combined treatment, either with 20 or 60 μM Rib. Since A → G and U → C are the most frequent mutation types associated with FU mutagenesis of LCMV and FMDV [35,36,37,38,39,71,90,92], this bias suggests a dominance of the mutagenic activity of FU even when Rib was present at a mutagenic concentration. The sequential treatment resulted in an increase of G → A transitions although the difference between the populations subjected to 20 and 60 μM Rib was not significant. In the sequential administration, when the medium with 60 μM Rib was removed to apply the medium containing FU, Rib might have reached a transient, intracellular mutagenic concentration, allowing viral replication and, as a consequence, an increased mutant spectrum complexity. Alternatively, 60 μM Rib may have a mutagenic activity as an intermediate mechanism before the inhibition is manifested. In our previous study on Rib mutagenesis [39] we did not distinguish whether the inhibitory activity of Rib was independent of its mutagenic activity or linked to it. It is possible that at early times after infection, the advantage of a sequential administration was not observed with 60 μM Rib because it was acting as a mutagen [74,75]. The possibility that high Rib concentrations might be inhibitory as a consequence of lethal mutagenesis of LCMV cannot be excluded, and it begs further investigations.

### 4.3. Implications and prospects for anti-arenavirus interventions

The results that we have reported here (Figure 1 and Table 2) have confirmed that Rib can be mutagenic for LCMV [39] and that a double mutagenic activity by Rib and FU was reflected in the mutant spectra of treated LCMV populations. Previous studies from our laboratory showed that a mutagenic activity can help extinguishing a virus resistant to another mutagen [93], a use of two mutagens that must be distinguished from their combined action. When present together, the outcome of two mutagens with different mutagenic specificities may be difficult to predict. Complex interactions have been previously described when two mutagenic activities (exogenous 5-azacytidine and endogenous APOBEC3G) acted on HIV-1 [94]. There is increasing evidence that the activity of two drugs often cannot be explained as the sum of activities of each drug when acting independently [74,75,95]. This fact, together with the dynamics of quasispecies replication (which involves changes in frequency of multiple subpopulations), should be considered in the design of antiviral strategies [15,74,75].

It may be viewed as premature that we emphasize the possibility of sequential inhibitor-mutagen protocols against arenavirus infections. At the time of this writing, a mutagenic activity of Rib against arenaviruses *in vivo* has not yet been demonstrated. However, animal model systems are available to test a possible mutagenic activity of Rib *in vivo*, and the search and design of antiviral base and nucleoside analogues is an expanding area of chemical pharmacology. In addition, several new non-mutagenic anti-arenavirus inhibitors are under investigation [11,12,13,96,97,98,99,100,101,13,96]. Thus, following the recent pioneer clinical trial with AIDS patients treated with a mutagenic pyrimidine analogue [51], arenaviruses offer a great potential for exploration of efficacy of alternative lethal mutagenesis protocols *in vivo*, while monitoring the composition of mutant spectra. This undertaking should be facilitated by the use of second- and third-generation massive sequencing techniques.

**Table 2:** Quasispecies analysis of LCMV populations in combined and sequential administration of Rib und FU.

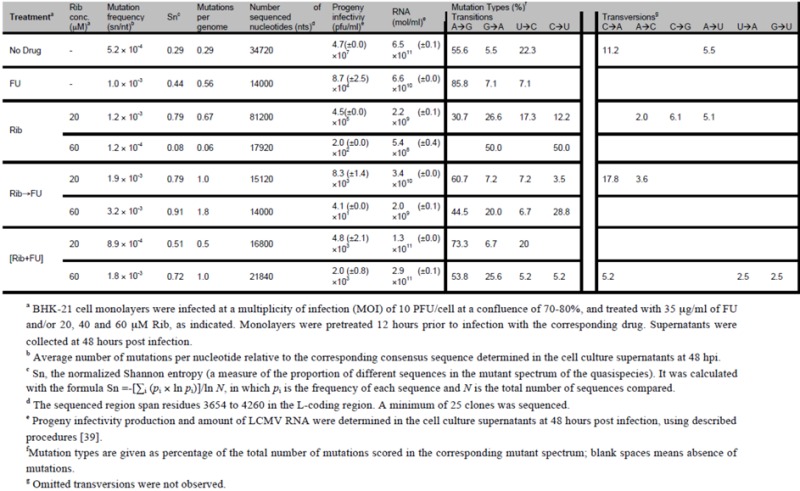

## 5. Concluding remarks

Quasispecies did not exist as a scientific concept until 1971, when Manfred Eigen published his pioneer treatise [102]. Since its completion by Eigen and Schuster [42], the theory has been extended to finite populations of replicons under non-equilibrium conditions, and several of its ground-breaking predictions (adaptability, limitations for sustained maintenance of information, ensembles of mutants as units of selection, *etc*.) have been applied and confirmed with complex biological entities such as the RNA viruses, with their highly compact genomes. Despite many unknowns in viral dynamics, the new insight provided by quasispecies is now permeating several aspects of RNA virus biology, including medical aspects such as the design of antiviral therapies. Treatment of arenavirus infections may also benefit from these developments.
